# Changes in *Mycoplasma pneumoniae* epidemiological and clinical features in children before, during, and after the COVID-19 pandemic in Shanghai, China

**DOI:** 10.1128/spectrum.02888-24

**Published:** 2025-08-07

**Authors:** Sheng Huang, Wenfang Zhuang, Ting Xu, Qikun Li, Liting Wu, Jia Li

**Affiliations:** 1 Department of Laboratory Medicine, Shanghai General Hospital, Shanghai Jiao Tong University School of Medicine 56694, Shanghai, China; 2 Medical Laboratory, Shidong Hospital Affiliated to University of Shanghai for Science and Technology, Shanghai, China; Kwame Nkrumah University of Science and Technology, Kumasi, Ghana

**Keywords:** *Mycoplasma pneumoniae*, children, COVID-19, epidemiological features, clinical features

## Abstract

Non-pharmaceutical interventions (NPIs) implemented during the COVID-19 pandemic had a significant impact on the epidemiology of respiratory pathogens. Following the relaxation of these measures, a notable increase in *Mycoplasma pneumoniae* (Mp) pneumonia incidence among children was observed. To comprehensively analyze the impact of NPIs on the patterns of Mp infections in children, we assessed changes in the epidemiology and clinical features of Mp infections in Songjiang District, Shanghai, before, during, and after the pandemic. A total of 6,050 hospitalized children with community-acquired pneumonia (CAP) from January 2017 to December 2024 were included. Annual Mp epidemiology was analyzed from 2017 to 2024. To assess NPI-specific effects, three comparative phases were defined: Before (June 2017–May 2019, pre-pandemic), During (June 2020–May 2022, active pandemic with NPIs), and After (June 2022–May 2024, post-NPIs). Epidemiological characteristics, clinical features, and laboratory findings were systematically compared across these phases. From 2017 to 2024, the number of hospitalized CAP cases significantly decreased from 2020 to 2022, with an overall Mp positivity rate of 43.16%. Annual Mp positivity rate peaked at 56.32% in 2022 versus 30.70% in 2020. Intergroup comparisons revealed that the After group exhibited the highest Mp positivity rate (56.03%), significantly exceeding both the Before (28.59%) and During (41.63%) groups. Notably, the After group exhibited significantly lower white blood cell count (WBC) and C-reactive protein (CRP), alongside elevated platelet count (PLT) and alanine aminotransferase (ALT). The proportion of patients with low Mp antibody titers (1:80) was significantly higher in the During group, whereas high-titer categories (1:320 and >1:640) predominated in the After group. NPIs during the COVID-19 pandemic significantly altered the epidemiology and clinical features of Mp infections in children in Songjiang District, Shanghai.

**IMPORTANCE:**

This study examines the effects of non-pharmaceutical interventions (NPIs) during the COVID-19 pandemic on the epidemiology of *Mycoplasma pneumoniae* (Mp) infections in children. By analyzing data from over 6,000 hospitalized children with community-acquired pneumonia, the research reveals a significant change in Mp infection patterns, with a marked increase in positivity rates after NPIs were relaxed. The findings indicate a shift in the affected demographic, with more school-age children presenting with Mp infections, as well as variations in clinical features such as different laboratory results. This research provides valuable insights for healthcare professionals in understanding the implications of NPIs on respiratory infections in children, guiding future treatment approaches and public health strategies. It represents a meaningful contribution to the ongoing evaluation of pediatric health in a post-pandemic context.

## INTRODUCTION

Community-acquired pneumonia (CAP) continues to be a leading cause of morbidity and mortality among children in developing countries (1, 2). Among the diverse etiological agents responsible for CAP, *Mycoplasma pneumoniae* (Mp) stands out as a significant pathogen. Children are particularly vulnerable to Mp infections, which can lead to severe clinical manifestations, including atypical pneumonia, respiratory distress, and prolonged hospitalization (3, 4). Furthermore, the alarming rise in antibiotic resistance rates complicates the management of Mp infections, as conventional therapeutic options become increasingly ineffective (5). The absence of a targeted vaccine against Mp adds to the urgency of understanding the epidemiology and clinical features of Mp pneumonia in children (4, 6, 7).

The COVID-19 pandemic has brought a shift in public health strategies worldwide, particularly through the implementation of non-pharmaceutical interventions (NPIs) (8–11). In China, the management of COVID-19 was elevated to a Class A infectious disease under the National Infectious Disease Prevention Law effective January 20, 2020. This designation facilitated the deployment of rigorous public health measures, including social distancing, mandatory mask-wearing, hand hygiene practices, and the closure of educational institutions, prompting a swift transition to online learning. These NPIs proved effective in limiting the transmission of the SARS-CoV-2 virus; however, they also had unforeseen consequences on the epidemiology of respiratory pathogens among children (12–15). As the COVID-19 pandemic transitioned into a new normal, the lifting of stringent public health measures raised concerns regarding the resurgence of respiratory infections, including those caused by Mp (16–18).

This study aims to elucidate the epidemiological trends and clinical characteristics of pediatric *Mycoplasma pneumoniae* pneumonia (MPP) in Shanghai, in the context of varying societal responses to the COVID-19 pandemic. By examining these attributes, we aim to provide valuable references for the diagnosis and prevention of pediatric CAP, thereby fostering enhanced awareness within society regarding the long-term implications of respiratory infections in children.

## MATERIALS AND METHODS

### Study population

This study conducted a retrospective analysis of children under 14 years old who were hospitalized for CAP at Shanghai General Hospital from January 1, 2017, to December 31, 2024. Diagnosis of CAP was made in accordance with Chinese guidelines and included clinical symptoms and signs consistent with pneumonia, as well as radiographic evidence of consolidation, infiltrate, or pleural effusion (Respiratory Group of Pediatric Branch of Chinese Medical Association, 2015). Patients with incomplete clinical data, those hospitalized for less than 1 day, and those who had underlying disease(s) such as malignant tumors, immunodeficiencies, and those receiving immunosuppressive therapy were excluded from the study. Demographic data and clinical information were collected from medical records. This study was reviewed and approved by the Institutional Review Board (IRB) of Shanghai General Hospital. The research protocol and the application for exemption of informed consent were evaluated and granted approval (Approval Letter: [2024-482]). All procedures and methodologies strictly adhered to relevant ethical guidelines and regulations. The exemption of informed consent was justified based on the nature of the research, which posed minimal risk to participants and did not involve the collection or publication of personally identifiable information. The age groups of the children were categorized as follows: infants (<1 year), toddlers (1–2 years), preschoolers (3–5 years), and school-age children (6–14 years).

### Case definitions

The diagnosis of MPP is based on the “Childhood *Mycoplasma pneumoniae* pneumonia diagnosis and treatment guidelines” (National Health Commission of the People’s Republic of China, 2023). These guidelines stipulate the presence of clinical symptoms, signs, and imaging findings characteristic of pneumonia, in addition to one or two of the following criteria: (i) a single serum Mp antibody titer of ≥1:160 (using the passive agglutination method) and (ii) positive Mp-DNA test result (polymerase chain reaction or sequencing technologies).

### Specimen collection and laboratory testing

Venous blood samples were collected using sterile techniques. For serum samples, a standard serum separator tube without anticoagulants was utilized, allowing the blood to clot at room temperature for 30 minutes prior to centrifugation. The samples were then centrifuged at 1,895 × *g* for 10 minutes to separate the serum. For whole blood samples, ethylenediaminetetraacetic acid (EDTA) or citrate anticoagulant tubes was used to prevent coagulation. Nasopharyngeal swabs were obtained by inserting a sterile swab into the nostril, gently advancing it until reaching the nasopharynx, and then rotating it for a few seconds to collect epithelial cells and secretions before being withdrawn. The swab was placed in a sterile container for transport to the laboratory.

The SERODIA-MYCO II diagnostic kit (Japan) was employed for semi-quantitative detection of Mp antibodies in human serum using the passive particle agglutination (PPA) method according to the manufacturer’s instructions. Mp DNA in nasopharyngeal swab was detected using an *in vitro* nucleic acid amplification kit (Fosun Diagnostics, Shanghai, China) following the manufacturer’s protocols. Nasopharyngeal swab samples were eluted in sterile saline solution. DNA was extracted using the kit-provided reagents and amplified on an ABI 7500 Real-time PCR system. A Ct value ≤38 with a characteristic S-shaped amplification curve was defined as positive. White blood cell count (WBC), C-reactive protein (CRP), and platelet count (PLT) were measured using an automated hematology analyzer (Mindray 5800, Shenzhen, China). Procalcitonin (PCT) levels were determined using a chemiluminescent immunoassay (Roche Cobas e 601 immunoassay analyzer). Alanine aminotransferase (ALT), lactate dehydrogenase (LDH), and Na^+^ were quantified using a clinical chemistry analyzer (Siemens Advia 2400 Chemistry System). D-Dimer levels were assessed on an automated coagulometer (Sysmex CA-7000), which allows for rapid detection and quantification of D-Dimer in plasma samples. All the reagents were provided by the respective manufacturers as part of pre-packaged kits, ensuring consistency and reliability across tests. All testing was performed in accordance with rigorous quality control protocols.

### Statistical analyses

Statistical analyses were performed using GraphPad Prism 8.2.1 software (La Jolla, CA, USA). Categorical variables were compared using the chi-square test and presented as counts (percentages). Continuous variables with non-normal distributions were compared using the Kruskal-Wallis test, followed by Dunn’s multiple comparisons, and reported as median (IQR; Q1–Q3). *P* < 0.05 was considered to be statistically significant (NS, not significant; *P < 0.05; **P < 0.01; ***P < 0.001; ****P < 0.0001).

## RESULTS

### Study population

A total of 6,050 children hospitalized for CAP from January 2017 to December 2024 were included, with the specific annual numbers detailed in Table 1. Compared to other years, the number of hospitalizations during the years 2020, 2021, and 2022 showed a significant decline, with the most pronounced decreases observed in 2020 and 2022 (*X^2^* = 838.57, *P* < 0.0001). Regarding the gender distribution among the children with CAP, there were 2,730 male and 3,320 female patients, indicating a significant difference in sex distribution from 2017 to 2024 (*X^2^* = 45.75, *P* < 0.0001). The analysis of within-age-group comparisons across years demonstrated that infants (0–1 year) exhibited the highest hospitalization rate in 2020 (*X^2^* = 340.20, *P* < 0.0001), toddlers (1–3 years) had the highest in 2021 (*X^2^* = 46.20, *P* < 0.0001), and school-age children (6–14 years) had the highest hospitalization rate in 2023 (*X^2^* = 561.40, *P* < 0.0001) (Table 1).

**TABLE 1:** Participant characteristics from 2017 to 2024^*a*^

Parameter	Value for year:								*X* ^2^	*P* value^*b*^
	2017	2018	2019	2020	2021	2022	2023	2024		
*n*	975	971	969	329	602	380	814	1,010	838.57	<0.0001
Gender, *n* (%)										
Male	397 (40.72%)	407 (41.92%)	445 (45.92%)	128 (38.91%)	282 (46.84%)	148 (38.94%)	412 (50.61%)	511 (50.59%)	45.75	<0.0001
Female	578 (59.28%)	564 (58.08%)	524 (54.08%)	201 (61.09%)	320 (53.16%)	232 (61.06%)	402 (49.39%)	499 (49.41%)		
Age, *n* (%)										
Infants	409 (41.95%)	384 (39.54%)	288 (29.72%)	180 (54.71%)	207 (34.38%)	105 (27.63%)	118 (14.49%)	201 (19.90%)	340.20	<0.0001
Toddlers	196 (20.10%)	235 (24.20%)	194 (20.02%)	55 (16.71%)	157 (26.07%)	75 (19.73%)	130 (15.97%)	160 (15.84%)	46.20	<0.0001
Preschoolers	260 (26.67%)	257 (26.46%)	278 (28.68%)	65 (19.75%)	172 (28.57%)	109 (28.68%)	232 (28.50%)	260 (25.74%)	13.40	0.063
School-age children	110 (11.28%)	95 (9.80%)	209 (21.58%)	29 (8.83%)	66 (10.98%)	91 (23.96%)	334 (41.04%)	389 (38.51%)	561.40	<0.0001

^*a*^ Infants, <1 year; toddlers, 1–2 years; preschoolers, 3-5 years; school-age children, 6–14 years.

^*b*^ Statistical comparisons across the years 2017–2024 were performed using the chi-square test, with results presented as frequency counts (percentages). A *P*-value of less than 0.05 was deemed statistically significant.

### Epidemiological characteristics of Mp over years

Among the 6,050 pediatric patients with CAP included in the study, a total of 2,611 cases tested positive for Mp, resulting in an overall positive rate of 43.16%. The highest annual Mp-positive rate was 56.32% in 2022, followed by 56.14% in 2024 and 52.70% in 2023 (*X^2^* = 344.60, *P* < 0.0001). The positive rate dropped to 30.70% in 2020, the first year of the COVID-19 pandemic. When stratified by age, it was observed that 2022 was the year with the highest positive rate for Mp across all age groups (Table 2).

**TABLE 2:** Distribution of *Mycoplasma pneumoniae*-positive specimens from 2017 to 2024^*a*^

Parameter	Value for year:								*X* ^2^	*P* value^*b*^
	2017	2018	2019	2020	2021	2022	2023	2024		
MP positive, *n*	297	251	471	101	281	214	429	567	344.60	<0.0001
Total positive rate	30.46%	25.85%	48.61%	30.70%	46.68%	56.32%	52.70%	56.14%		
Infants	24/409 (5.86%)	14/384 (3.64%)	27/288 (9.37%)	7/180 (3.88%)	29/207 (14.00%)	15/105 (14.28%)	11/118 (9.32%)	17/201 (8.46%)	34.58	<0.0001
Toddlers	82/196 (41.83%)	66/235 (28.08%)	103/194 (53.09%)	33/55 (60.00%)	98/157 (62.42%)	48/75 (64.00%)	69/130 (53.07%)	84/160 (52.50%)	68.76	<0.0001
Preschoolers	126/260 (48.46%)	113/257 (43.96%)	179/278 (64.38%)	41/65 (63.07%)	106/172 (61.62%)	78/109 (71.55%)	125/232 (54.31%)	160/260 (61.54%)	46.68	<0.0001
School-age children	65/110 (59.09%)	58/95 (61.05%)	162/209 (77.51%)	20/29 (68.96%)	48/66 (72.72%)	73/91 (80.21%)	224/334 (67.03%)	306/389 (78.66%)	33.84	<0.0001

^*a*^ Infants, <1 year; toddlers, 1–2 years; preschoolers, 3–5 years; school-age children, 6–14 years.

^*b*^ Statistical comparisons across the years 2017–2024 were performed using the chi-square test, with results presented as frequency counts (percentages). A *P*-value of less than 0.05 was deemed statistically significant.

### Epidemiological characteristics of Mp before, during, and after the COVID-19 pandemic

Since the outbreak of the COVID-19 pandemic, various regions in China have implemented a series of control measures, including maintaining social distancing, wearing masks, working from home, and closing schools to conduct online education. These measures reduced opportunities for virus transmission and potentially altered the interactions and competition among respiratory pathogens. To further investigate the epidemiological characteristics of Mp before, during, and after the COVID-19 pandemic in the Shanghai area, we strategically selected a representative subset of 72 months (24 months per group) from the overall data set. This selection was based on Shanghai’s pandemic control timeline (notably the full lifting of restrictions on June 1, 2022) (19, 20), ensuring equivalent 24-month observation windows with synchronized seasonal coverage across all groups. The three-phase categorization was defined as follows: the Before group (hospital admissions from June 1, 2017, to May 31, 2019), the During group (hospital admissions from June 1, 2020, to May 31, 2022), and the After group (hospital admissions from June 1, 2022, to May 31, 2024).

The study documented 1,945, 968, and 1,508 pediatric CAP hospitalizations in the Before, During, and After groups, respectively, with the During group exhibiting a statistically significant reduction in admissions compared to both Before and After groups (*X^2^* = 487.62, *P* < 0.0001). Among the four age groups, the infant (0–1 year; *X^2^* = 197.50, *P* < 0.0001) and toddler (1–3 years, *X^2^* = 27.08, *P* < 0.0001) groups had the highest proportion of CAP patients in the During period, while school-age children (6–14 years) comprised the majority in the After period (*X^2^* = 453.80, *P* < 0.0001) (Table 3).

**TABLE 3:** Participant characteristics before, during, and after the COVID-19 pandemic^*a*^

Parameter	Value for group:			*X* ^2^	*P* value^*e*^
	Before^*b*^	During^*c*^	After^*d*^		
*n*	1,945	968	1,508	487.62	<0.0001
Gender, *n* (%)					
Male	827 (42.52%)	427 (44.11%)	729 (48.34%)	11.92	0.0026
Female	1,118 (57.48%)	541 (55.89%)	779 (51.66%)		
Age, *n* (%)					
Infants	753 (38.71%)	379 (39.15%)	273 (18.10%)	197.50	<0.0001
Toddlers	443 (22.78%)	230 (23.76%)	248 (16.45%)	27.08	<0.0001
Preschoolers	512 (26.32%)	266 (27.48%)	405 (26.86%)	0.45	0.7979
School-age children	237 (12.19%)	93 (9.61%)	582 (38.59%)	453.80	<0.0001

^*a*^ Infants, <1 year; toddlers, 1–2 years; preschoolers, 3–5 years; school-age children, 6–14 years.

^*b*^ Before group: hospital admissions from 1 June 2017 to 31 May 2019.

^*c*^ During group: hospital admissions from 1 June 2020 to 31 May 2022.

^*d*^ After group: hospital admissions from 1 June 2022 to 31 May 2024.

^*e*^ The chi-square test was employed for statistical analysis, and the results were expressed as counts (percentages). A *P*-value of less than 0.05 was deemed statistically significant.

**Fig 1:**
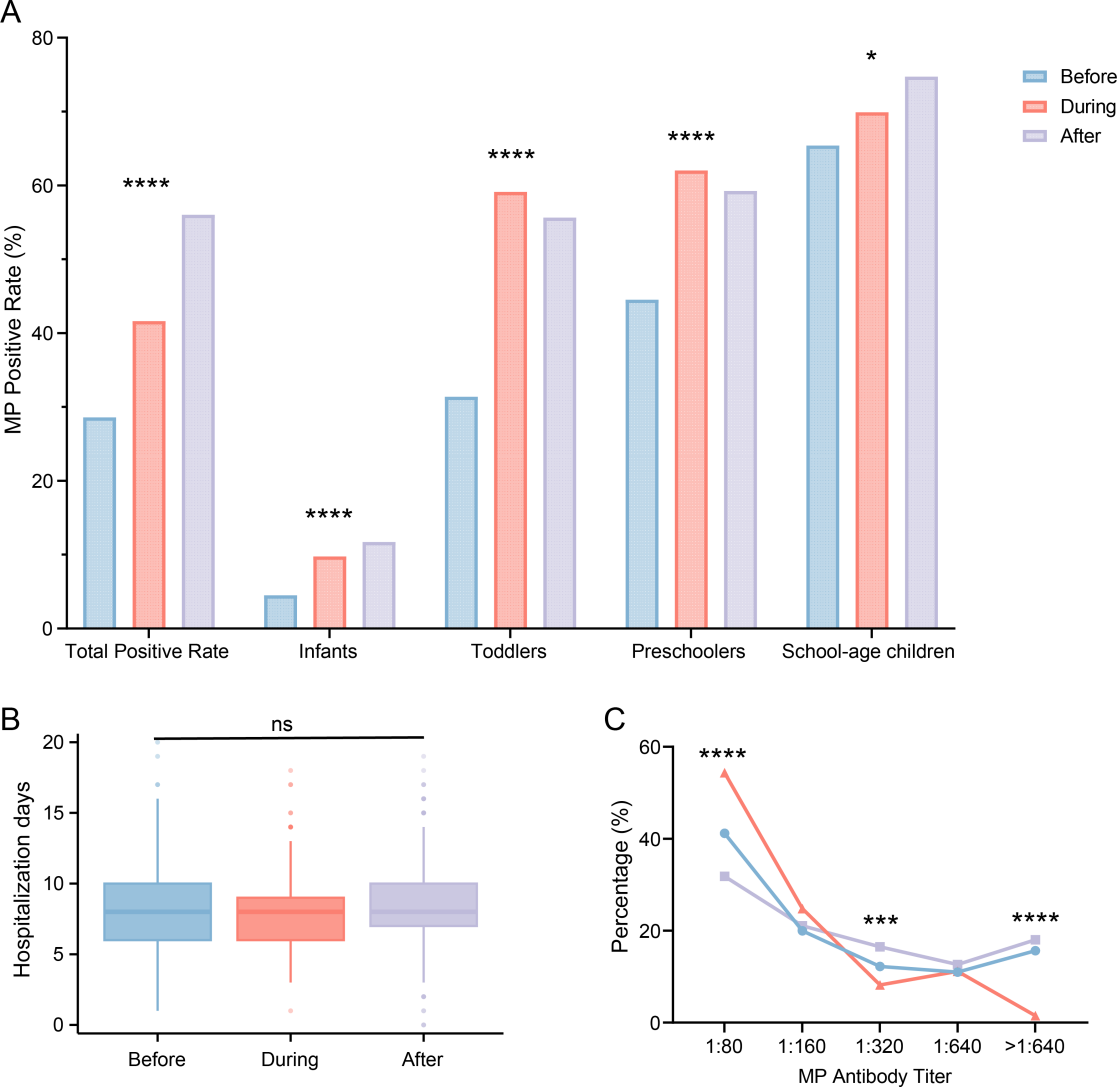
The effects of the COVID-19 pandemic on Mp-positive rates and clinical characteristics of Mp-positive patients. (**A**) Mp positivity rates across pediatric age groups during different phases of the COVID-19 pandemic. Before group (*n* = 1945): Comprises hospital admissions between June 1, 2017, and May 31, 2019. During the group (*n* = 968): Comprises hospital admissions between June 1, 2020, and May 31, 2022. After group (*n* = 1508): Comprises hospital admissions between June 1, 2022, and May 31, 2024. “Total” represents 0–14 years. Then, the study population was stratified into four age groups: infants (<1 year), toddlers (1–2 years), preschoolers (3–5 years), and school-age children (6–14 years). Positivity rates are expressed as percentages (%). Intergroup comparisons were analyzed using Pearson’s chi-square test. The asterisk (*) indicates statistical significance across the three groups. (**B**) Hospitalization days of Mp-positive patients across Before (*n* = 556), During (*n* = 403), and After (*n* = 845) groups. Data were analyzed using the Kruskal-Wallis test, followed by Dunn’s multiple comparisons. ns denotes no statistically significant differences among the three groups. (**C**) Comparative analysis of Mp antibody titer in Mp-positive patients across Before (*n* = 556), During (*n* = 403), and After (*n* = 783) groups. The chi-square test was employed for statistical analysis, and the results were expressed as percentages (%). A *P*-value of less than 0.05 was deemed statistically significant. *P < 0.05; **P < 0.01; ***P < 0.001; ****P < 0.0001.

The overall Mp-positive rates in hospitalized children with CAP were 28.59%, 41.63%, and 56.03% in the Before, During, and After groups, respectively (Fig. 1A and Table 4). The overall Mp-positive rate in the After group was significantly higher than those in both Before and During groups (Fig. 1A and Table 4, *X^2^* = 265.30, *P* < 0.0001). Regarding the age distribution of Mp cases, the infants group (0–1 year) and school-age group (6–14 years) exhibited the highest positive rate in the After period (infants: *X^2^* = 19.83, *P* < 0.0001; school-age: *X^2^* = 7.452, *P* = 0.0241), while the other two age groups (toddlers: 1–3 years and preschoolers: 3–6 years) showed the highest positive rates in the During period compared to the Before and After periods (toddlers: *X^2^* = 63.16, *P* < 0.0001; preschoolers: *X^2^* = 29.74, *P* < 0.0001) (Fig. 1A and Table 4).

**TABLE 4:** Distribution of *Mycoplasma pneumoniae*-positive specimens before, during, and after the COVID-19 pandemic^*a*^

Parameter	Value for group:			*X* ^2^	*P* value^*e*^
	Before^*b*^	During^*c*^	After^*d*^		
MP positive, *n*	556	403	845	265.30	<0.0001
Total positive rate	28.59%	41.63%	56.03%		
Infants	34/753 (4.52%)	37/379 (9.76%)	32/273 (11.72%)	19.83	<0.0001
Toddlers	139/443 (31.38%)	136/230 (59.13%)	138/248 (55.65%)	63.16	<0.0001
Preschoolers	228/512 (44.53%)	165/266 (62.03%)	240/405 (59.26%)	29.74	<0.0001
School-age children	155/237 (65.40%)	65/93 (69.89%)	435/582 (74.74%)	7.452	0.0241

^*a*^ Infants, <1 year; toddlers, 1–2 years; preschoolers, 3–5 years; school-age children, 6–14 years.

^*b*^ Before group: hospital admissions from 1 June 2017 to 31 May 2019.

^*c*^ During group: hospital admissions from 1 June 2020 to 31 May 2022.

^*d*^ After group: hospital admissions from 1 June 2022 to 31 May 2024.

^*e*^ The chi-square test was employed for statistical analysis, and the results were expressed as counts (percentages). A *P*-value of less than 0.05 was deemed statistically significant.

### Effects of the COVID-19 pandemic on the clinical characteristics of Mp-positive patients

The utilization rate of bronchoalveolar lavage (BAL) in Mp-positive children exhibited no statistically significant differences among the three groups (Table 5, *X^2^* = 2.81, *P* = 0.2451). Similarly, the incidence of pleural effusion (*X^2^* = 1.20, *P* = 0.5484) and duration of hospitalization (Kruskal-Wallis *H* = 5.90, *P* = 0.0523) revealed comparable outcomes across all study periods (Table 5 and Fig. 1B). In addition, we analyzed Mp antibody titers among the three groups. The proportion of patients with low Mp antibody titers (1:80) was significantly higher in the During group (*X^2^* = 56.80, *P* < 0.0001), whereas the proportions of patients with high antibody titers (1:320, *X^2^* = 16.62, *P* = 0.0002 and >1:640, *X^2^* = 65.88, *P* < 0.0001) were markedly elevated in the After group (Fig. 1C and Table 5).

**TABLE 5:** Clinical characteristics of MP-positive patients before, during, and after the COVID-19 pandemic^*a*^

Parameter	Value for group:			*X*^2^ or *H*	*P* value^*e*^
	Before^*b*^	During^*c*^	After^*d*^		
Bronchoalveolar lavage	25/556 (4.50%)	27/403 (6.70%)	54/845 (6.39%)	2.81	0.2451
Pleural effusion	10/281 (3.56%)	7/187 (3.74%)	29/577 (5.03%)	1.20	0.5484
Hospitalization days, M (Q₁, Q₃)	8 (6,10)	8 (6, 9)	8 (7,10)	5.90	0.0523
MP antibody titer					
1:80	229/556 (41.19%)	219/403 (54.34%)	249/783 (31.80%)	56.80	<0.0001
1:160	111/556 (19.96%)	100/403 (24.81%)	165/783 (21.07%)	3.47	0.1767
1:320	68/556 (12.23%)	33/403 (8.19%)	129/783 (16.48%)	16.62	0.0002
1:640	61/556 (10.97%)	45/403 (11.17%)	99/783 (12.64%)	1.06	0.589
>1:640	87/556 (15.65%)	6/403 (1.49%)	141/783 (18.01%)	65.88	<0.0001

^*a*^ Infants, <1 year; toddlers, 1–2 years; preschoolers, 3–5 years; school-age children, 6–14 years.

^*b*^ Before group: hospital admissions from 1 June 2017 to 31 May 2019.

^*c*^ During group: hospital admissions from 1 June 2020 to 31 May 2022.

^*d*^ After group: hospital admissions from 1 June 2022 to 31 May 2024.

^*e*^ Categorical variables were compared using the chi square test and presented as counts (percentages). For continuous variables with non-normal distributions (e.g., hospitalization days), the Kruskal-Wallis test was employed, followed by Dunn’s multiple comparisons, with results reported as median (interquartile range; IQR; Q1–Q3). A *P-*value of less than 0.05 was deemed statistically significant.

### Effects of the COVID-19 pandemic on laboratory findings of Mp-positive patients

**Fig 2:**
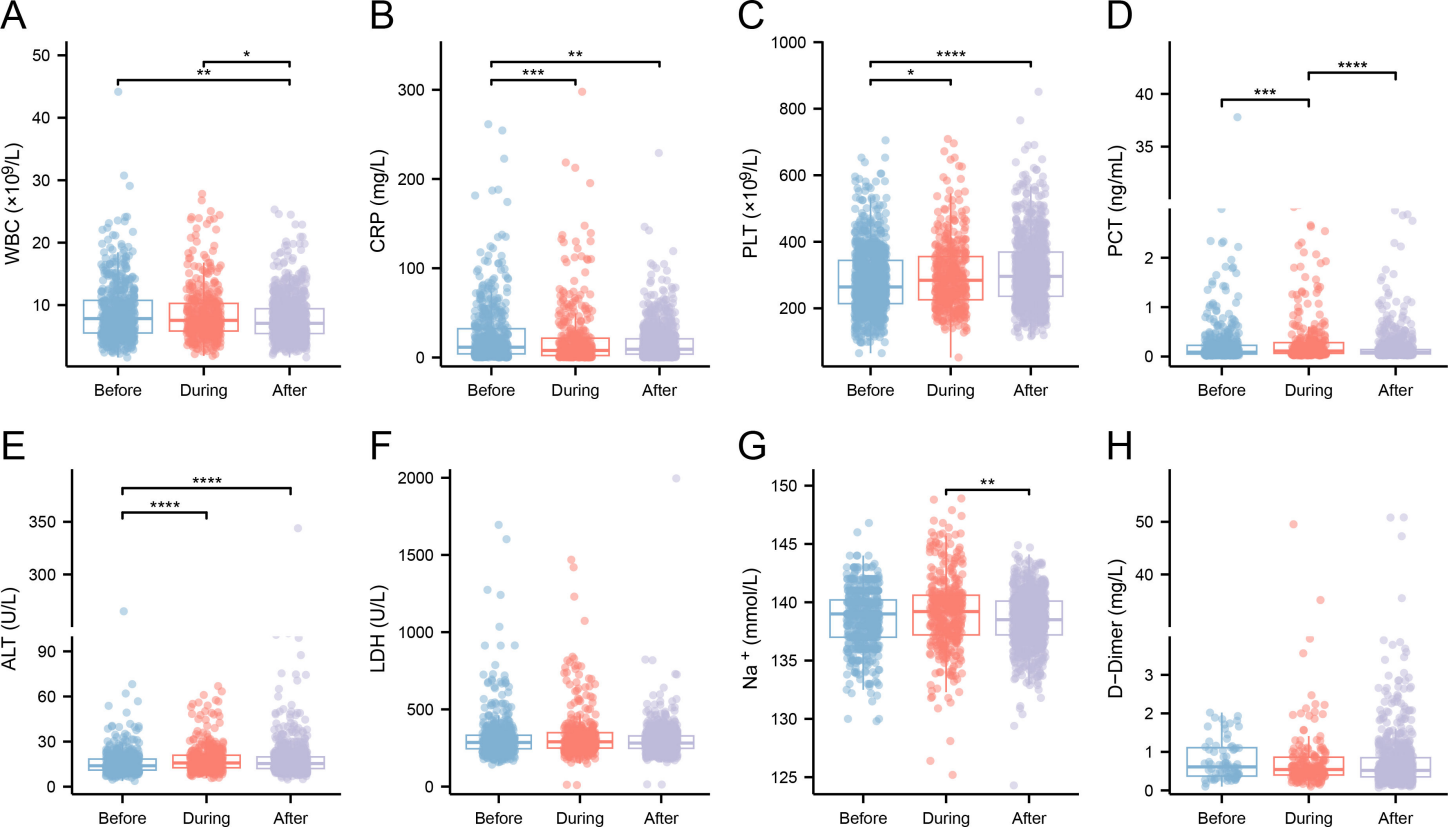
The effects of the COVID-19 pandemic on laboratory findings of Mp-positive patients. Comparative analysis of (**A**) WBC, (**B**) CRP, (**C**) PLT, (**D**) PCT, (**E**) ALT, (**F**) LDH, (**G**) Na^+^, and (**H**) D-Dimer levels in Mp-positive patients across Before, During, and After groups. Before group: Comprises hospital admissions between June 1, 2017, and May 31, 2019. During the group: Comprises hospital admissions between June 1, 2020, and May 31, 2022. After group: Comprises hospital admissions between June 1, 2022, and May 31, 2024. Data were compared using the Kruskal-Wallis test, followed by Dunn’s multiple comparisons. A *P*-value of less than 0.05 was deemed statistically significant. *P < 0.05; **P < 0.01; ***P < 0.001; ****P < 0.0001. WBC, white blood cell count. CRP, C-reactive protein. PLT, platelet count. PCT, procalcitonin. ALT, alanine aminotransferase. LDH, lactate dehydrogenase.

As presented in Fig. 2A and B, compared with the Before group, the After group showed significantly lower WBC and CRP. Conversely, PLT and ALT were markedly elevated in the After group compared to the Before group (Fig. 2C and E). Notably, PCT displayed a distinct temporal pattern, with the During group exhibiting significantly higher concentrations compared to both Before and After groups (Fig. 2D). Serum Na^+^ levels in the After group were significantly lower compared to the During group (Fig. 2G). No significant intergroup differences were observed in LDH or D-dimer levels across the three groups (Fig. 2F and H).

## DISCUSSION

Following the end of the COVID-19 pandemic period, there have been alarming reports from various regions indicating a significant rise in the incidence of Mp pneumonia among children (21–23). Alongside this increase, both the rates of antibiotic resistance and the incidence of severe cases have shown a troubling upward trend (24–26). Therefore, this study retrospectively analyzed the effect of the COVID-19 pandemic on epidemiological and clinical features of Mp infection using clinical real-world data in the Shanghai area. We seek to offer valuable data to provide a basis for the diagnosis and treatment of children affected by CAP in the post-COVID era.

The COVID-19 pandemic has significantly impacted the epidemiological landscape of various respiratory infections in children, including CAP caused by Mp. In China, particularly in Shanghai, the trajectory of the pandemic from 2017 to 2024 has been marked by varying public health interventions. Initially, in 2020, stringent control measures were enacted in response to COVID-19, characterized by strict lockdowns, travel restrictions, and mandatory mask-wearing, which markedly curtailed the circulation of respiratory pathogens. Between 2021 and 2022, these measures fluctuated, with periods of relaxation followed by reinstituted restrictions as case numbers rose and fell. Notably, mid-2022 to 2024 saw a complete reopening of public spaces and a return to normalcy, emphasizing the importance of this phase in evaluating the resurgence of respiratory infections. These varying interventions created a unique natural experiment, allowing us to observe the impact of public health measures on respiratory pathogen transmission.

In our study, we observed that the number of children admitted for CAP significantly declined during the pandemic years, with particularly notable reductions in 2020 and 2022. The Mp-positive rate showed a remarkable fluctuation, dropping to 30.70% in 2020 before reaching a peak of 56.32% in 2022, followed by 56.14% in 2024 and 52.70% in 2023. Analysis by age revealed that 2022 had the highest Mp-positive rate across all age groups. This pattern suggests a potential “rebound effect” in Mp infections following the relaxation of COVID-19 control measures. This significant variation in infection rates likely reflects the combined effect of multiple factors. First, the implementation of stringent NPIs, particularly social isolation measures, may have contributed to diminished herd immunity through reduced population exposure to the pathogen, thereby creating an “immunity gap” (27). This phenomenon likely stems from diminished immunological priming in pediatric populations during prolonged periods of low pathogen circulation. Second, as the prevalence of COVID-19 diminished, Mp may have gained an advantage in the ecological niche competition among respiratory pathogens. In addition, increased vigilance toward respiratory symptoms post-pandemic may have led to higher detection rates (28), partially explaining the observed increase in infection rates. Our results align with data from another study conducted in Shanghai during the same timeframe (16), which reported an average Mp DNA positive rate of 28.21% from 2014 to 2018, dropping to 1.61% during strict epidemic control, and then surging to 35.96% in 2023. These trends are further supported by a German multicenter study analyzing 38,204 patients from 2015 to 2024, which also revealed a significant post-COVID-19 pandemic surge in outpatient Mp cases (29). In addition, a nationwide population-based cohort study in Denmark, including 14,241 children and adolescents with a positive Mp PCR test from 2016 to 2024, demonstrated a 2.9-fold rise in Mp infections and a 2.6-fold increase in hospitalizations in 2023–2024 compared to pre-COVID-19 seasons (30). The stark contrast between the low rates during strict control measures and the subsequent surge highlights the complex interplay between public health interventions and pathogen dynamics.

To further analyze the impact of COVID-19 prevention policies on CAP and Mp-positive rates in pediatric patients, this study employs a stratified design to explore the impact of public health interventions on the transmission of Mp. To ensure the scientific rigor of temporal comparisons, a three-phase observational framework was established: referencing the milestone event of Shanghai’s pandemic control (the full lifting of lockdown on 1 June 2022), the phases include (i) the Before group (June 2017–May 2019), reflecting the natural transmission state pre-pandemic; (ii) the During group (June 2020–May 2022), characterizing the effects of high-intensity non-pharmaceutical interventions (NPIs); and (iii) the After group (June 2022–May 2024), assessing post-pandemic transmission characteristics. To control for seasonal confounding, each group incorporated 24 consecutive months of data (spanning two complete epidemiological years), with a standardized observation window (June to May annually) to eliminate potential climatic interference on respiratory infection rates. This methodological design effectively distinguishes the causal effects of NPIs from natural fluctuations, providing a temporal comparison model to dissect the triadic dynamics of policy-pathogen-host interactions.

Our analysis revealed a significant shift in the age distribution of CAP patients, with infants predominating in the Before and During groups, while school-age children became the majority in the After group. The overall Mp-positive rate in the After group (56.03%) was markedly higher than in the Before (28.59%) and During (41.63%) groups, with increases observed across all age groups. The observed shift in age distribution among pediatric patients holds significant implications for clinical management and public health strategies. This alteration may be attributed to several interrelated factors. The resumption of in-person schooling has likely facilitated the transmission of Mp among school-age children, primarily due to increased close contact. Moreover, the immune systems of the school-age group could be more susceptible to sudden exposure following a prolonged period of isolation. Changes in children’s social behavior post-pandemic may also contribute to an elevated risk of infection. These hypotheses necessitate further investigation to elucidate their validity and implications for pediatric health outcomes.

In our study, no significant differences were observed in the proportions of BAL treatment, incidence of pleural effusion, or length of hospital stay among the three groups. However, we observed that the During group had the highest proportion of patients with low Mp antibody titers (1:80), whereas the After group exhibited a significant increase in children with high titers (1:320 and >1:640). This shift can be explained by several interrelated factors, including increased antigen exposure due to the resumption of social activities such as school reopenings and gatherings, which likely triggered a stronger immune response. In addition, the “immunity gap” caused by reduced natural exposure to pathogens during the pandemic may have led to more intense immune reactions when pathogens re-emerged. Furthermore, potential mutations or increased virulence of Mp could have elicited a more robust immune response, resulting in higher antibody titers. Collectively, these factors provide a plausible explanation for the observed rise in high antibody titers among children in the After group.

Laboratory findings revealed that levels of WBC and CRP in the After group were significantly lower than those in the Before group. This may be attributed to the control of infection following treatment of hospitalized children, which could have led to a corresponding decrease in the activation of the immune response. Conversely, ALT levels in the After group were significantly higher than those in the Before group. There have been reports of liver injury as a complication of Mp infection; however, the specific mechanisms remain to be elucidated, as both innate and acquired immunity are believed to be involved (31). It is important to note that our findings did not exclude potential confounders influencing ALT elevation, including drug-induced liver injury and comorbid viral infections (e.g., hepatitis viruses). Future studies will focus on systematic liver function monitoring to further clarify causality. In addition, the PLT levels in the After group were higher than those in the other groups. Juan Qiu et al. reported that, compared to patients with non-severe *Mycoplasma pneumoniae* pneumonia (NSMPP), those with severe *Mycoplasma pneumoniae* pneumonia (SMPP) exhibited significantly elevated PLT levels (32). However, in our study, there was no significant difference in disease severity among the Before, During, and After groups. This discrepancy suggests that elevated PLT levels may reflect broader systemic inflammatory responses rather than solely indicating disease severity. Further research is needed to investigate the relationship between PLT levels, inflammatory markers, and clinical outcomes in Mp-infected children. The observed changes in laboratory parameters underscore the need for comprehensive monitoring of children with Mp infections, particularly in the post-pandemic era, to better understand the evolving clinical manifestations and optimize treatment strategies.

This study has several limitations that warrant consideration. First, prior to the COVID-19 pandemic, the clinical implementation rate of antibody and nucleic acid testing for respiratory pathogens was significantly lower than that observed post-pandemic, which may lead to an underestimation of the data in the Before group. Second, the 8-year retrospective design, while providing longitudinal insights, introduces potential confounding from evolving healthcare practices over the extended observation period. Third, antibiotic resistance testing was not routinely performed on all samples, which limits our ability to assess the impact of resistance patterns on clinical outcomes and epidemiological trends. Nevertheless, through a retrospective analysis of data from hospitalized children with CAP at a large comprehensive hospital, this study provides a comprehensive perspective on Mp infections in children before, during, and after the COVID-19 pandemic in Shanghai. We elucidated the gradual resurgence and epidemic patterns of Mp and compared the clinical characteristics of affected children across the different epidemic phases. These findings offer important references for the prevention and treatment of pediatric Mp infections.
